# Transporter NRT1.5/NPF7.3 suppresses primary root growth under low K^+^ stress by regulating the degradation of PIN-FORMED2

**DOI:** 10.1186/s12870-022-03730-6

**Published:** 2022-07-08

**Authors:** Youyou Wang, Ran Wang, Shuang Zhao, Changmei Lu, Ziqiang Zhu, Hong Li

**Affiliations:** Jiangsu Key Laboratory for Biodiversity and Biotechnology, College of Life Sciences, Nanjing Normal University, Nanjing, 210023 China

**Keywords:** NRT1.5, Low potassium stress, Primary root growth, Auxin signaling, PIN2

## Abstract

**Background:**

The availability of potassium is one of the main environmental factors for modifying the plasticity of root architecture. Many potassium channels and transporters are involved in regulating primary root growth in response to low potassium stress. NRT1.5/NPF7.3 transporter is a NO_3_^−^/H^+^ and K^+^/H^+^ cotransporter, and participates in NO_3_^−^ and K^+^ translocation from the roots to the shoots. However, the underlying mechanism of NRT1.5-regulated primary root growth under low potassium stress is unclear.

**Results:**

We show that NRT1.5/NPF7.3 inhibited primary root growth under low potassium conditions by regulating the accumulation of PIN2 protein and auxin levels. Under low potassium conditions, the mutants *nrt1.5* and *lks2* exhibited longer primary roots, longer meristem regions and elongation zones of primary roots, and more cell activity in the meristem region compared to WT plants, revealing the involvement of NRT1.5 in LK (low potassium)-inhibition primary root growth. In addition, exogenous auxin (IAA), auxin analogue (NAA, 2.4-D) or auxin precursor (IBA) promoted the primary root growth of WT and the complementation line NRT1.5 COM plants. In addition, the application of NPA inhibited the primary root growth of the *nrt1.5* and *lks2* mutants. Auxin accumulation was higher in the root tip of *nrt1.5* plants than in WT plants, indicating that NRT1.5 regulates root growth inhibition by regulating auxin distribution. Furthermore, PIN2 was degraded more quickly in *nrt1.5* plants under LK stress.

**Conclusions:**

Our findings reveal that NRT1.5 inhibits primary root growth by modulating the auxin level in the root tip via the degradation of PIN2.

**Supplementary Information:**

The online version contains supplementary material available at 10.1186/s12870-022-03730-6.

## Background

As sessile organisms, plants have to properly modify their organ development to enhance their ability to capture resources. In particular, the plasticity of root architecture is a highly adaptive response that enables plants to counteract the spatial and temporal changes in the availability of water and mineral ions with different foraging strategies [1, 2]. Nutrient availability can exert a profound impact on root architecture. When grown under limited phosphorus availability, plants show inhibition of primary root elongation and increase the initiation of lateral roots [3–5]. In contrast to low phosphorus stress, low nitrogen promotes the growth of primary roots and lateral roots [3, 6].

Potassium (K^+^), the most abundant cation in living plant cells, plays an essential role in plant growth and development [7]. However, the K^+^ concentration in soils is low and fluctuates [8, 9]. In addition, the availability of K^+^ in soils is spatially heterogeneous. Therefore, plants must modulate their root architecture to forge potassium. In *Arabidopsis thaliana*, K^+^ deficiency impairs primary root and lateral root development while inducing root hair elongation [10, 11]. Interestingly, diverse species of plants show different responses to K^+^ deficiency [12–14]. For example, low potassium stress reduces the lateral root elongation of *Arabidopsis* (Col-0) [12, 13], but increases the overall lateral root growth of barley seedlings [14].

The phytohormone auxin plays crucial roles in plant responses to K^+^ deficiency [15, 16]. The auxin level of roots is downregulated under LK conditions [17]. The K^+^ transporters KUP2/6/8 regulate lateral root formation by modulating auxin signalling [18]. In addition, the detection of *ProDR5*:*GUS* expression in roots showed that LK stress induces auxin redistribution in primary roots [19], indicating that auxin distribution is essential for root growth in response to LK stress. Moreover, KUP4 and KUP9, two members of the K^+^ transporter KUP/HAK family, function as auxin transporters [19–21]. The K^+^ transporter KUP4/TRH1 regulates root hair development and root gravitropism by modulating auxin transport [19, 22]. KUP9 mediates auxin efflux from the ER lumen to the cytoplasm, and regulates primary root growth in response to low potassium stress [21].

Auxin transport in roots through influx and efflux carriers (such as AUX1 and PINs) promotes auxin-dependent root growth in response to potassium deficiency. Auxin is mobilized via AUX1, AXR1, and AXR2 to root tips to inhibit primary root growth under LK conditions [23]. The inhibition of primary root growth by K^+^ deficiency can be reversed in auxin-resistant mutants [23]. Auxin transport towards the root tips is maintained by basally localized PIN1, PIN3 and PIN7 in the stele [24]. PIN2 localizes at the epidermal and cortex cells of root tips and mediates the upward and downward flow of auxin from the root tip towards the elongation zone, creating an auxin gradient in roots together with other transporting proteins [25, 26]. The accumulation and subcellular localization of PIN proteins are essential for the formation of the auxin gradient, which is important for the root development response to different stresses [22, 27, 28]. Previous studies have indicated that potassium transporter proteins participate in regulating the accumulation and subcellular localization of PIN proteins. KUP4/TRH1 regulates the distribution of the auxin efflux carrier PIN1 [19, 22, 27]. In addition, the potassium channel AKT1 regulates K^+^-dependent root growth by modulating auxin transporter PIN1 degradation and auxin redistribution in the root [28]. PIN2 is involved in root hair growth and primary root growth after LP or Peps (plant elicitor peptides) treatment [29, 30]. Whether PIN2 functions in primary root growth under potassium deficiency is still unclear.

NRT1.5/NPF7.3 acts as a NO_3_^−^/H^+^ and K^+^/H^+^ cotransporter, and participates in NO_3_^−^ and K^+^ translocation from the roots to the shoots [31, 32]. NRT1.5 also functions as the transporter of IBA, the precursor of auxin indole-3-acetic acid (IAA), and regulates root gravitropism [33]. NRT1.5/NPF7.3 belongs to the NRT1/PTR family, which contains as many as 53 members. Some members of this family have been shown to participate in regulating phytohormone-mediated plant growth and development [34–36]. NRT1.1 plays a key role in triggering the stimulation of LR elongation through auxin-signalling pathways [36]. NPF3 can mediate GA transmembrane transportation, and participates in seed germination, hypocotyl, and primary root growth [34]. NRT1.2 can act as an ABA transporter to regulate root growth [35].

NRT1.5 has been reported to transport IBA and regulate the synthesis of auxin [33]. Here we demonstrated that NRT1.5 has a new function in regulating auxin distribution. Our results showed that NRT1.5 regulated the degradation of PIN2 and promoted upward auxin flow, resulting in a decrease in auxin accumulation in root tips, thereby suppressing root growth under LK conditions.

## Results

### NRT1.5 participates in LK-mediated inhibition of primary root growth

The *nrt1.5* mutant maintains primary root growth under low K^+^ stress. NRT1.5, one of the most important transporters in *Arabidopsis*, conducts K^+^ and NO_3_^−^ translocation from the roots to the shoots [31, 32]. Our previous report showed that the *nrt1.5* mutant displays a phenotype sensitive to low K^+^ stress, and exhibits chlorosis of the leaves on LK medium [32]. However, it is interesting that the primary root of the *nrt1.5* mutant continues to grow, but the wild-type root stops growing after transfer from MS to LK medium [32]. This result suggested that NRT1.5 may play an important role in regulating primary root growth under low potassium stress. In this study, we explored the mechanism of NRT1.5 in regulating primary root growth under low potassium stress.

**Fig. 1:**
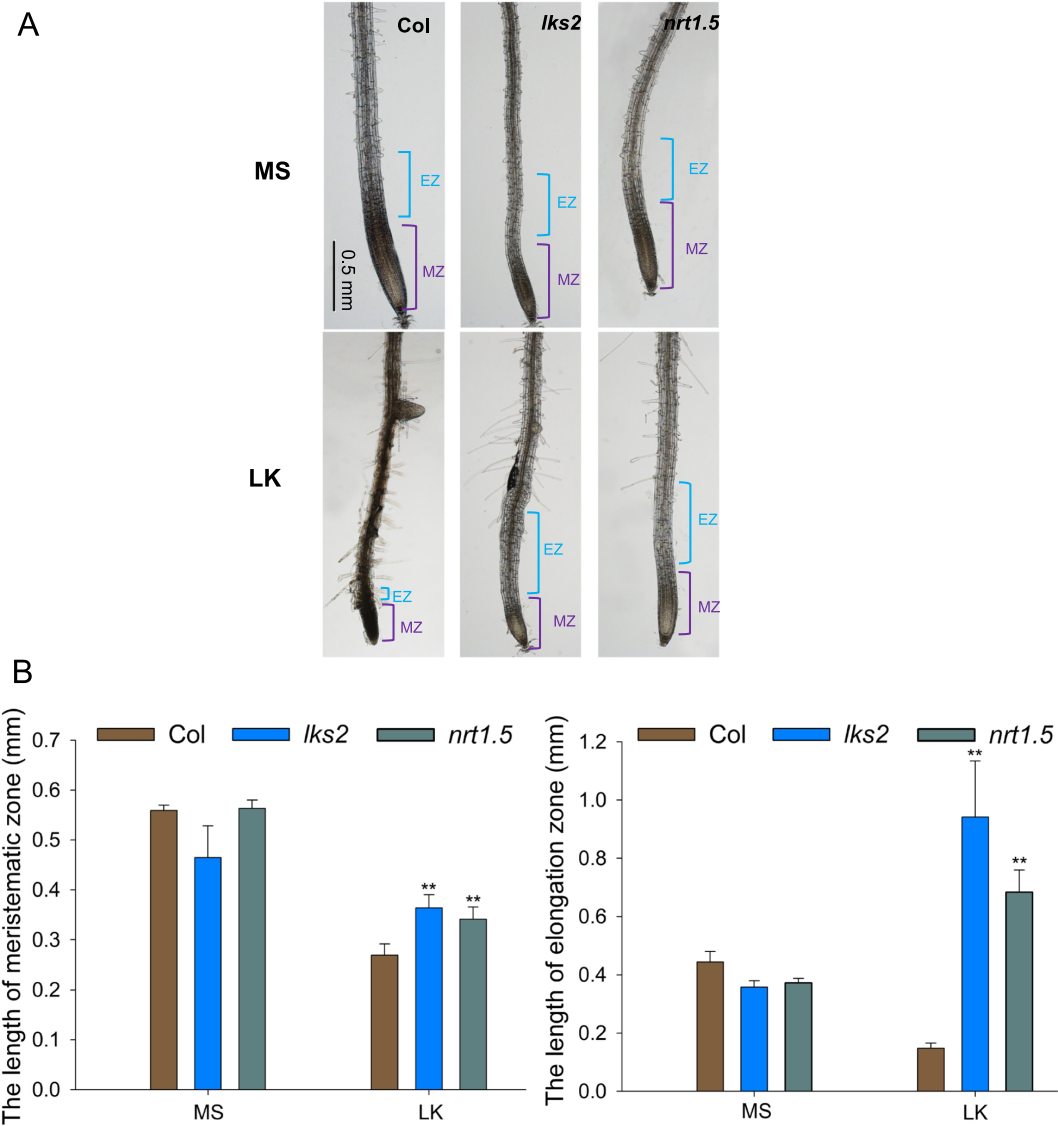
The *nrt1.5* mutant possesses a longer meristem zone and elongation zone under low potassium stress. **A** Phenotype comparison of meristem zone and elongation zone length in wild-type *Arabidopsis* (Col), *nrt1.5* and *lks2*. Seeds were germinated on MS medium for 4 days, and then transferred to MS or LK (low K^+^, 0.1 mmol/L) medium for 3 days, bar = 0.5 mm. **B** The lengths of the meristem zone and elongation zone shown in (**A**). Data are shown as means ± SE (*n* = 20). Student’s *t*-test (* *P* < 0.05; ** *P* < 0.01) was used to analyze statistical significance

Both cell division in the meristem zone (MZ) and cell elongation in the elongation zone (EZ) contribute to root growth. To evaluate the role of NRT1.5 in LK-mediated primary root inhibition, we initially observed and measured the meristem zone and elongation zone of primary roots under LK stress. It has been reported that LKS2 is a point-mutational form of NRT1.5 [32]. Therefore, the *lks2* (point mutation) and *nrt1.5* (T-DNA insertion) mutants were used in this study. We transferred 4-d-old seedlings to LK medium or MS medium for 3 days. In MS medium, there was no obvious difference in the length of MZ and EZ between mutants (*nrt1.5* and *lks2*) and wild-type plants (Fig. 1). However, under LK stress, the meristem zone and elongation zone in the *lks2* and *nrt1.5* mutants were longer than those in the wild-type plants (Fig. 1). It is worth noting that the meristem of wild-type became dark after a period of growth in LK medium. The meristem is the source of all root cells [37, 38]. The short and dark meristem zones in wild-type plants suggest that meristem activity may be impaired in wild-type plants under LK conditions. To explore the cell activity in various plants under LK conditions, we finally examined the cell activity by trypan blue staining. The results showed that cells in the MZ of the wild-type and NRT1.5 COM plants were stained by trypan blue after 3 days of low potassium treatment, while the cells in *lks2* and *nrt1.5* plants were not stained (Fig. S1), indicating that cell death occurred in the MZ of wild-type plants after low potassium treatment, which was consistent with the dark root tip of wild-type (Fig. 1).

**Fig. 2:**
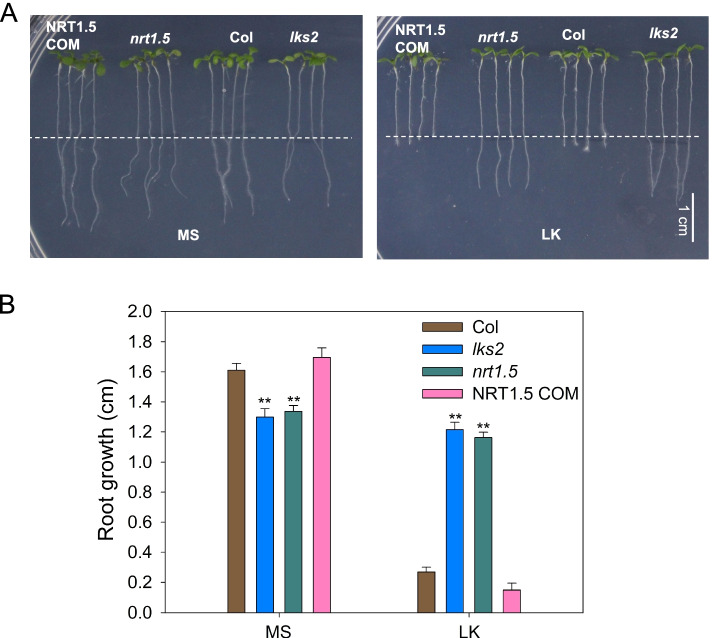
The *nrt1.5* and *lks2* mutant maintains primary root growth on low K^+^ medium. **A** Root growth phenotype of wild-type *Arabidopsis* (Col), *nrt1.5* and *lks2* and the *nrt1.5* complementation line NRT1.5 COM under low potassium stress. Seeds were germinated on MS medium for 4 days, and then the seedlings were transferred to MS or LK (low K^+^, 0.1 mmol/L) medium for 3 days, bar = 1 cm. **B** The lengths of the primary root growth shown in (**A**). Data are shown as means ± SE (*n* = 20). Student’s *t*-test (* *P* < 0.05; ** *P* < 0.01) was used to analyze statistical significance

Then we measured the growth of primary root in various plants after LK treatment for 3 days. We transferred 4-d-old seedlings to LK medium and measured the primary root growth after 3-day treatment. We found that the primary root growth of wild-type plants was blocked in LK medium (Fig. 2). However, the primary roots of the *lks2* and *nrt1.5* mutants continued growing under LK stress (Fig. 2). The complementation line NRT1.5 COM was able to rescue the primary root growth of *nrt1.5* plants to wild-type plant levels (Fig. 2). Taken together, these results suggest that NRT1.5 repress the growth of primary roots by regulating the cell activity, cell division, cell elongation in the root tip response to LK stress.

### Auxin participates in NRT1.5-suppressed root growth

**Fig. 3:**
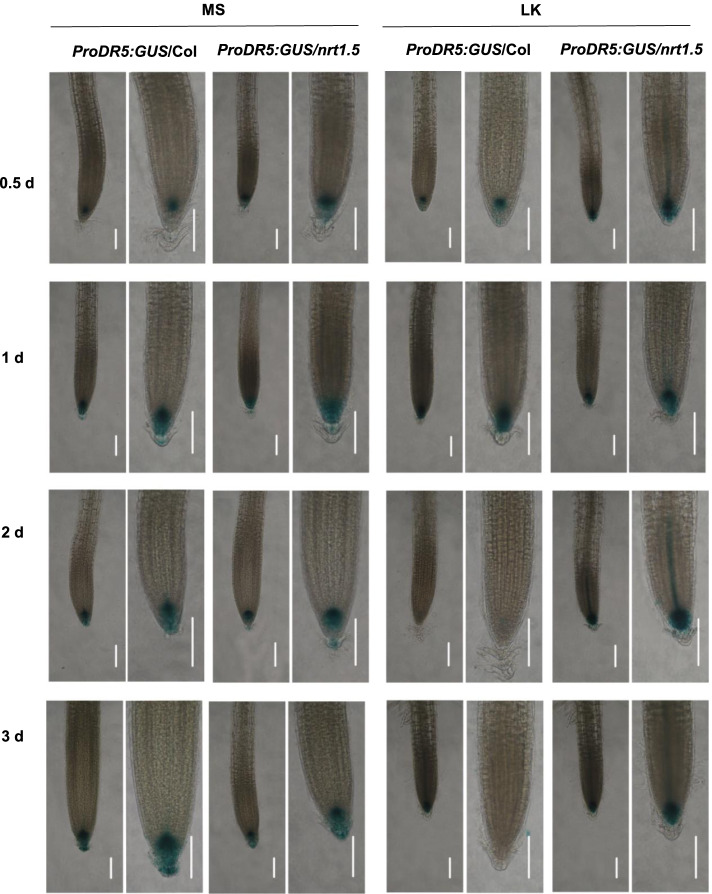
The *nrt1.5* mutant possesses a higher auxin concentration in the primary root tip. The 4-d-old seedlings of the *ProDR5*:*GUS* crossing line were transferred to MS and LK media for different days and then stained to detect GUS expression, bars = 0.1 mm

The biosynthesis and transport of the phytohormone auxin are critical in regulating primary root growth in response to LK. We speculated that NRT1.5 may regulate primary root growth through auxin signalling. To monitor the auxin distribution in roots after LK treatment, a biosensor *ProDR5:GUS* line [39] containing *DIRECT REPEAT 5* (*DR5*)-driven GUS was crossed with the *nrt1.5* mutant. As shown in Fig. 3, there was no obvious difference in the GUS signals between wild-type and the *nrt1.5* mutant after transferred to MS or LK medium for 0.5 days and 1 day (Fig. 3). But the GUS signals in the wild-type root tips was significantly declined after transferred to LK medium for 2 days and 3 days, while these signals in the *nrt1.5* root tips were stable (Fig. 3). The results indicated that the *nrt1.5* mutant possessed a higher auxin concentration in the primary root tip than wild-type under LK stress, which was consistent with the root growth phenotype (Fig. 2). These data confirm that NRT1.5 regulates primary root growth through regulating the accumulation or distribution of auxin.

**Fig. 4:**
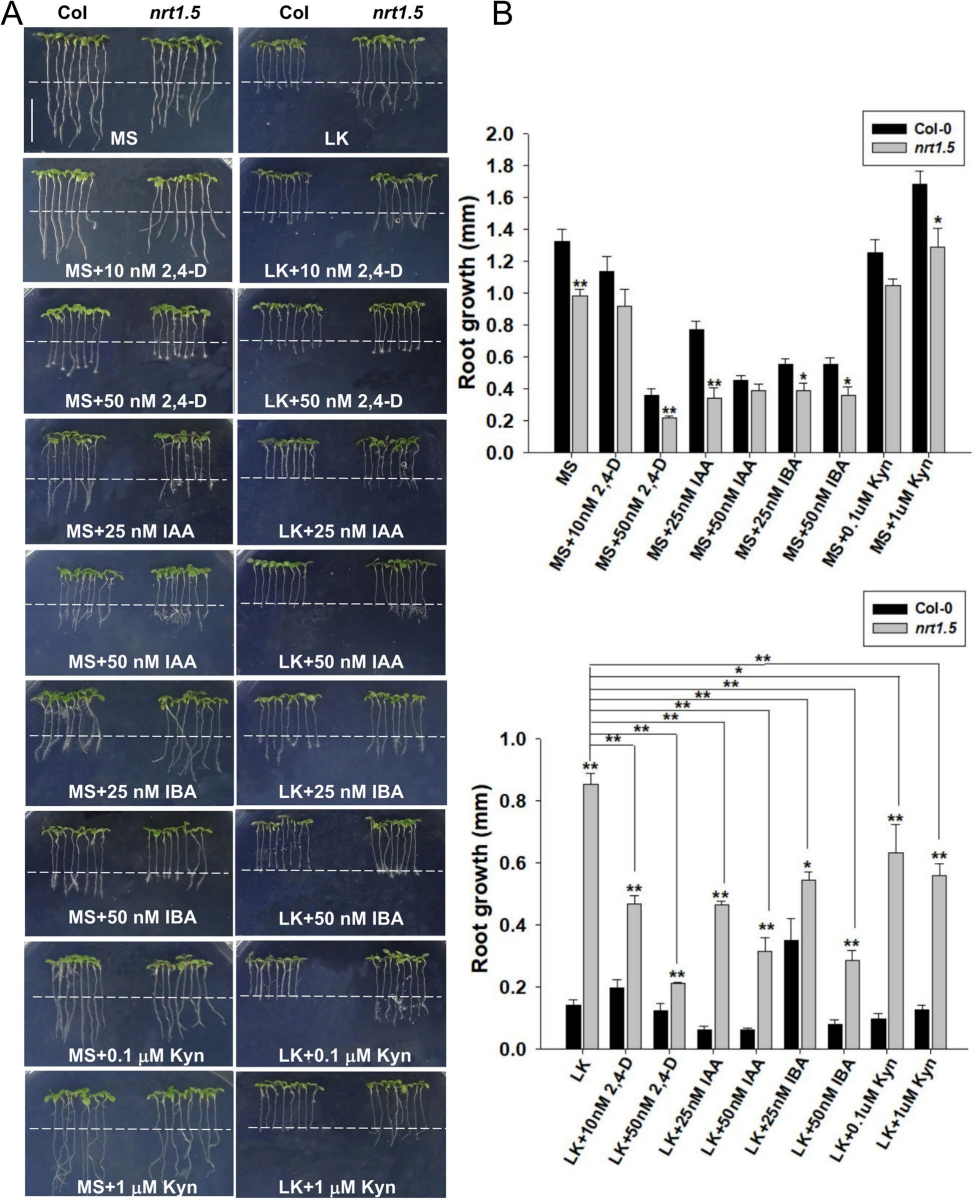
NRT1.5-regulated root growth is dependent on auxin accumulation. **A** Root growth of seedlings (Col, *nrt1.5*) under MS or LK medium containing auxins (IAA, 2,4-D), auxin biosynthesis precursor (IBA), and auxin biosynthesis inhibitor (Kyn). Four-day-old seedlings were transferred to MS or LK medium containing 2.4-D (10 nM, 50 nM), IAA (25 nM, 50 nM), IBA (25 nM, 50 nM) and Kyn (0.1 μM, 1 μM) for 3 days, bar = 1 cm. **B** The lengths of the primary root growth shown in (**A**). Data are shown as means ± SE (*n* = 20). Student’s *t*-test (* *P* < 0.05; ** *P* < 0.01) was used to analyze statistical significance

**Fig. 5:**
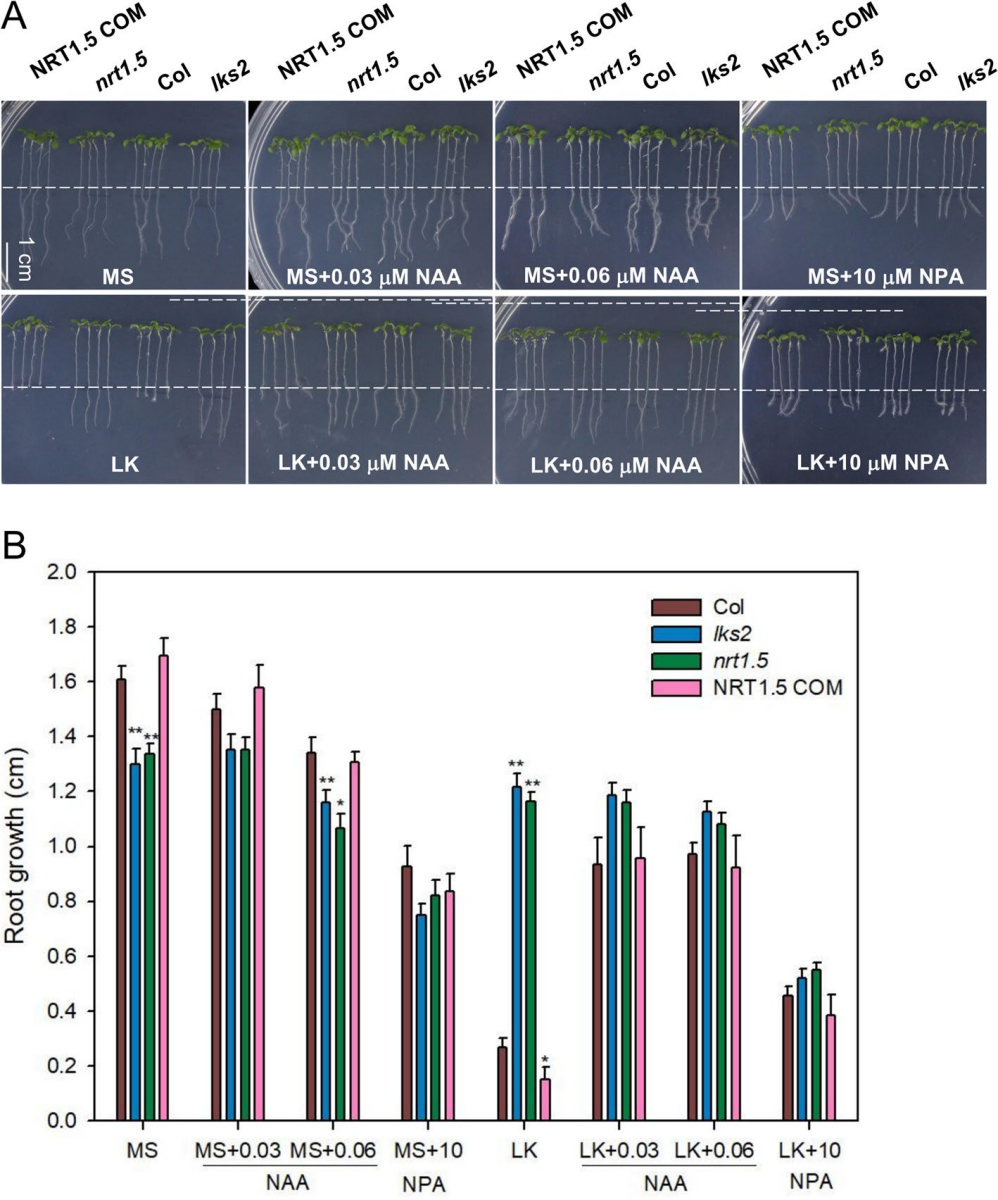
NRT1.5-inhibited root growth is dependent on auxin polar transport. **A** Root growth of seedlings (Col, *nrt1.5*, *lks2*, and the *nrt1.5* complementation line NRT1.5 COM) under MS or LK medium containing auxin analogue NAA or auxin polar transport inhibitor NPA. Four-day-old seedlings were transferred to MS or LK medium containing 30 nM NAA, 60 nM NAA or 10 μM NPA for 3 days, bar = 1 cm. **B** The lengths of the primary root growth shown in (**A**). Data are shown as means ± SE (*n* = 20). Student’s *t*-test (* *P* < 0.05; ** *P* < 0.01) was used to analyze statistical significance

To verify whether the long-root phenotype of *nrt1.5* mutants is dependent on auxin, we treated plants with endogenous auxin IAA (indole-3-acrtic acid), its synthesis precursor IBA (indole-3-butyric acid), and synthetic auxin analogue 2.4-D. We found that both IAA and 2.4-D dramatically inhibited root growth *nrt1.5* mutant, but didn’t significantly inhibit root growth in Col (Fig. 4). Interestingly, the addition of 25 nM IBA significantly enhanced the wild-type root growth, and inhibited root growth in *nrt1.5* seedlings. Higher doses of IBA at concentrations more than 50 nM dramatically inhibited *nrt1.5* root growth, but did not significantly enhanced wild-type root growth (Fig. 4), suggesting that NRT1.5-regulated primary root growth is related to auxin concentration. Therefore, we blocked auxin biosynthesis with TAA1/TAR inhibitor Kyn (L-kynurenine), and found that Kyn also have an inhibitory effect on *nrt1.5* mutant root growth, we also added the auxin NAA (1-naphthaleneacetic acid) into MS and LK medium. After addition of NAA into the LK medium, primary root growth was affected. On LK medium, exogenous NAA promoted the primary roots of the wild-type and the complementation line plants, but there was no effect on the primary roots of the *nrt1.5* and *lks2* plants (Fig. 5). Moreover, the auxin transport inhibitor NPA (N-1-naphtylphthalamic acid) inhibited the primary root growth of *nrt1.5* and *lks2* (Fig. 5), further supporting that our hypothesis that NRT1.5 regulates primary root growth by regulating auxin accumulation.

We assumed that auxin signalling is involved in NRT1.5-regulated primary root growth under LK conditions. The application of auxins increased the auxin concentration in both wild-type and *nrt1.5* seedlings primary root. As auxins regulate root growth in a dose-dependent manner, the increase of auxin concentration promoted root growth in wild-type, but inhibited the root growth of *nrt1.5* mutant, which is consistent with the expression of *ProDR5:GUS*. Furthermore, both auxin polar transport inhibitor (NPA) and auxin biosynthesis inhibitor (Kyn) inhibited the primary root growth of *nrt1.5* plants, further confirming the hypothesis that NRT1.5-inhibited primary root growth is dependent on auxin.

### NRT1.5 reduces PIN2 abundance under LK stress

**Fig. 6:**
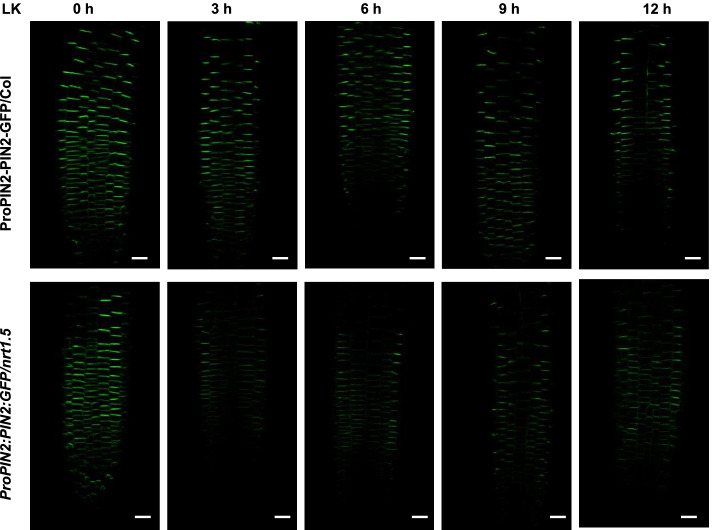
NRT1.5 promotes the abundance of PIN2 under LK stress. The *ProPIN2:PIN2-GFP* crossed lines were transferred to LK (0.1 mmol/L K^+^) medium for 3 h, 6 h, 9 h and 12 h. GFP fluorescence in plant roots was observed at the indicated time points, bars = 50 μm

The local auxin concentration in the root apex is highly determined by polar auxin transport. In *Arabidopsis*, PIN family proteins, especially PIN1, PIN2, PIN3, PIN4, and PIN7, are components of the auxin efflux machinery that precisely mediate polar auxin transport and function in roots [40–43]. Given that the auxin level in *nrt1.5* root tips is higher than that in Col root tips, we reasoned that NRT1.5 might modify polar auxin transport in the root under LK stress. To test this idea, we examined the expression and protein abundance of PINs. First, we analyzed the mRNA levels of *PIN2* and *PIN3* in *nrt1.5* and wild-type plants. The results showed that the *PIN2* transcript in the wild-type roots was markedly induced when plants were transferred from MS to LK medium (Fig. S2). However, the *PIN2* transcript in the *nrt1.5* mutant was weakly induced after LK treatment (Fig. S2). RT-qPCR analysis revealed that the *PIN3* transcript in wild-type and *nrt1.5* plants was inhibited. In addition, the *PIN3* transcript in *nrt1.5* plants were higher than that in wild-type in both MS plants and LK media (Fig. S2). Second, we crossed *ProPIN2:PIN2-GFP* or *ProPIN3:PIN3-GFP* with *nrt1.5* plants to obtain the *ProPIN2:PIN2-GFP*/*nrt1.5* and *ProPIN3:PIN3-GFP*/*nrt1.5* plants and examined the distribution and abundance of PIN2 and PIN3 under low potassium stress. We observed similar PIN2 fluorescence distribution patterns in wild-type and *nrt1.5* roots before LK treatment (Fig. 6). We then examined the effect of low potassium stress on PIN2-GFP abundance in the *nrt1.5* mutant and wild-type plants. The levels of PIN2-GFP fluorescent signals in wild-type roots were comparable within 6 h of LK treatment. After 9 h of LK treatment, the PIN2-GFP signals were slightly decreased in wild-type plants. However, PIN2 levels in *nrt1.5* mutants obviously decreased within 3 h of LK treatment (Fig. 6). The PIN2-GFP signals in *nrt1.5* plants maintained a very low level within 6 h, 9 h, and 12 h of LK treatment (Fig. 6). When compared with wild-type plants, *nrt1.5* showed weaker PIN2-GFP fluorescent signals under LK stress. The plasma membrane (PM)-located PIN2 proteins cycle between the PM and endosomal compartments, which in turn determines the direction and activity of auxin transport [44]. To explore whether NRT1.5 may alter the subcellular localization of PIN2, a more-detailed view of PIN2-GFP was observed after LK treatment for 3 h and 6 h. As shown in Fig. S3, PIN2-GFP abundance was reduced after LK treatment in *nrt1.5* mutant, but PIN2-GFP-labeled endosomal compartments wasn’t detected. We also examined similar PIN3-GFP fluorescence distribution patterns in wild-type and *nrt1.5* roots under MS and LK medium (Fig. S4). These results showed that the lack of NRT1.5 decreased PIN2 accumulation, implying that NRT1.5 plays a negative role in regulating the degradation of PIN2 under LK stress.

## Discussion

The availability of mineral nutrients in the soil is heterogeneous, which is one of the main factors limiting crop production. To capture more resources for high crop production, plant roots have to respond to nutritional status. The root system architectural response is one of the most important strategies for adapting to fluctuating nutrient availability in the soil [1, 2]. To date, we have a detailed understanding of the effects of nutrients, especially low nutrient stresses, on primary root growth [3, 4, 28]. In *Arabidopsis*, low Pi and low K^+^ stress results in a reduction in primary root length, while low N stress enhances primary root growth [3, 4, 28]. In this study, we found that low potassium inhibited primary root growth, which is consistent with a previous report [28].

NRT1.5 is an important transporter that translocates NO_3_^−^ and K^+^ from the roots to the shoots, regulating tolerance to stresses (salt, drought and cadmium), and lateral root development [31, 32, 45, 46]. Our data indicate that NRT1.5 has a major role in regulating primary root growth under low potassium stress, leading to a decrease in primary root growth. Both mutants (*nrt1.5* and *lks2*) showed strongly increased primary root growth under low potassium conditions (Fig. 2). Moreover, the length of the meristem zone and elongation zone were strongly increased in the *nrt1.5* mutants (Fig. 1), which indicated that NRT1.5 regulated cell division and elongation under low potassium stress.

Auxin is a key regulator of root plastic growth, and its abundance decreases in the root tip under potassium deficiency [28]. External application of NAA significantly alleviated LK-induced root growth inhibition (Fig. 5), indicating that the reduction in auxin levels in the root tip is the main reason for primary root growth inhibition. Moreover, primary root growth is affected by polar auxin transport under low potassium conditions. Previous studies revealed that potassium transporters are involved in auxin-regulated root growth by regulating auxin redistribution and the auxin response [18, 20, 27, 28]. TRH1/KUP4 may possess auxin transport activity, and regulates localization of the auxin efflux carrier PIN1, root hair development and root gravitropism [19, 20, 27]. KUP2/6/8 regulate the auxin response and lateral root formation [18]. KUP9 functions as an auxin efflux carrier in QC cells and regulates root growth under low potassium stress [21]. AKT1 was reported to be required for the regulation of K^+^-dependent root growth by modulating PIN1 degradation and auxin redistribution in the root [28]. Here, we demonstrated that NRT1.5 may alleviate the degradation of PIN2 under LK, maintained upward auxin recycling, and decreased the auxin level in wild-type roots in LK medium. LK treatment induced the degradation of PIN2 in *nrt1.5* roots. The degradation of PIN2 decreased the increase in auxin flow and maintained auxin levels for root growth, resulting in primary root growth under low potassium. These results indicated that NRT1.5 may regulate auxin redistribution in primary roots under LK stress. Moreover, NRT1.5 directly mediates indole-3-butyric acid (IBA), a precursor of IAA, in yeast [33]. These data suggest that KUP4, KUP9, AKT1, and NRT1.5 coordinately control *Arabidopsis* root growth by regulating auxin homeostasis.

Local auxin distribution is achieved by the distinct localization of PIN proteins, which dynamically change in response to developmental cues, such as nutrient levels of the soil. PIN2, which localizes at the cortex and in epidermal cells, channels auxin flow from the root tip towards the elongation zone, creating the auxin gradient in roots together with other auxin transporting proteins [5, 47, 48]. Auxin efflux carriers PIN2 is important for modulation of root system architecture in response to different nutrient levels of the soil. In *Arabidopsis thaliana*, phosphorus deficiency promotes root hair number and length via suppressing vacuolar degradation of PIN2, thus enhancing root absorption capacity and efficiency [29]. PIN2 also plays key role in regulating primary and lateral root growth in response to nitrate, as nitrate-treated *pin2* mutants showed longer primary roots and less lateral roots number than wild-type plants [48]. Furthermore, the phosphorylation status of PIN2, which regulates polar plasma membrane localization, is necessary for root system architecture modulation in response to nitrate. Here, we found that the degradation of PIN2 was more quickly in *nrt1.5* mutant than wild-type under LK condition (Fig. 6), suggesting NRT1.5 may regulate the accumulation of PIN2 protein, accelerated the upward flow of auxin to the elongation zone, and blocked the auxin accumulation in the root tips.

In this study, we demonstrated the crucial roles of the transporter NRT1.5 in regulating primary root growth in response to LK stress. A mechanistic study indicated that NRT1.5 slowed the degradation of the auxin efflux carrier PIN2 and decreased auxin levels in the root tips.

## Materials and methods

### Plant materials and growth conditions

All the *Arabidopsis thaliana* seeds described in this manuscript were in Columbia (Col) ecotype. The seeds of Col, *nrt1.5* [32], *lks2* [32], *ProDR5*:*GUS* [39], *ProPIN2:PIN2-GFP* [49] and *ProPIN3:PIN3-GFP* [41] were obtained from Professor Weihua Wu’ Lab at China Agricultural University. The Col, *nrt1.5* (SALK_043036) were originally from the Arabidopsis Biological Resource Center (ABRC, OhioState University, Columbus, OH, USA). The *lks2* mutant was isolated from EMS-mutagenized Col, from Professor Weihua Wu’ Lab at China Agricultural University. The *ProDR5*:*GUS* is originally from Dr. Alonso at North Carolina State University, and *ProPIN2:PIN2-GFP* and *ProPIN3:PIN3-GFP* were originally from Dr. Scheres at Utrecht University. For generating *ProDR5*:*GUS*/*nrt1.5*, *ProPIN2:PIN2-*GFP/*nrt1.5* and *ProPIN3:PIN3-*GFP/*nrt1.5*, the *ProDR5*:*GUS*, *ProPIN2:PIN2-GFP and ProPIN3:PIN3-GFP* reporter lines were genetically crossed with *nrt1.5*, respectively. The homozygous lines were identified by PCR and GFP fluorescence or GUS observation. Although newly hybrid plant materials are not deposited in publicly seed platform, they are available for other researchers from the corresponding author on reasonable request. And plants are used complying with relevant and national regulations.

Seeds were initially surface sterilized with 6% (v/v) of NaClO and 0.1% (v/v) Triton X-100 for 10 min and then washed with sterile water for five times. After stratification at 4 °C for 2 days in the dark, the sterilized seeds were sown on Murashige and Skoog (MS) medium with 0.8% agar and 3% (w/v) sucrose, and germinated at 22 °C under constant illumination at 80–90 μmol m^− 2^ s^− 1^ for 3 days. The LK medium was prepared by modification of MS medium described previously [50]. KNO_3_ and KH_2_PO_4_ in MS medium were replaced by NH_4_NO_3_ and NH_4_H_2_PO_4_, respectively. So, the LK medium contained (in mM): 28.74 NH_4_NO_3_, 1.5 MgSO_4_·7H_2_O, 1.5 NH_4_H_2_PO_4_, 3 CaCl_2_·2H_2_O. The potassium almost came from agar in the LK medium.

For seed harvest and hybridization, *Arabidopsis* seedlings were grown in potting soil mixture (rich soil: vermiculite = 2:1, v/v) and kept in growth chambers at 22 °C with illumination at 120 μmol m^− 2^ s^− 1^ (16 h light/8 h dark period). The relative humidity was approximately 70% (±5%).

### Root growth phenotype analyses

The seeds were germinated and grown vertically on MS medium for 4 days, and then transferred to MS or LK medium with or without the chemicals. In order to prepare MS or LK medium with different concentrations (10 nM 2.4-D, 50 nM 2.4-D, 25 nM IAA, 50 nM IAA, 25 nM IBA, 50 nM IBA, 0.1 μM Kyn, 1 μM Kyn, 30 nM NAA, 60 nM NAA) of chemicals, first prepare stock solutions (10 μM IAA, 100 μM 2,4-D, 50 μM IBA, 5 mM Kyn, and 100 μM NAA). Then corresponding volumes of stock solutions were added into medium until the medium was sterilized by autoclaving at 121 °C for 20 min and cooled to 60 °C.

The photographs were taken after transferring for 3 days. The length of primary root growth was measured with software Image J. Experiments were performed three times. Data were from 20 plants and presented as the mean ± SE. The student’s *t* test was used to analyze statistical significance. Asterisks in the figures denote significant differences: *P < 0.05, **P < 0.01.

For measuring the length of the meristem zone (MZ) and the elongation zone (EZ), the primary roots were imaged under a dissecting microscope (Nikon) equipped with a Canon DSLR camera. Three independent repetitious were performed. The student’s *t* test was used to analyze statistical significance. Asterisks in the figures denote significant differences: *P < 0.05, **P < 0.01.

### GUS staining

After transferred to MS or LK medium for 3 days, the GUS reporter lines were incubated with GUS staining solution (1 mg mL^− 1^ 5-bromo-4-chloro-3-indolyl-β-D-glucuronic acid, 0.1% Triton X-100, 10 mM EDTA, 2 mM potassium ferrocyanide, 2 mM potassium ferricyanide, and 50 mM sodium phosphate buffer, pH 7.2) for 3 hours. After staining, the seedlings were washed with 75% ethanol and then imaged under a dissecting microscope (Nikon) equipped with a Canon DSLR camera.

### Trypan blue staining

For trypan blue staining, 4-day-old seedlings were transferred onto MS or LK medium for 3 days. The roots were stained with 0.4% Trypan blue for 5 min, rinsed 3 times, and photographed under a dissecting microscope (Nikon) equipped with a Canon DSLR camera.

### Confocal laser scanning microscopy

After germination on MS medium for 4 days, the seedlings of GFP crossing lines were transferred to MS or LK medium for 3 h, 6 h, 9 h and 12 h. Images were captured on the confocal laser scanning microscope (Carl Zeiss LSM710 META). Scanner and detector settings were optimized to avoid saturation and to maximize resolution and kept unchanged throughout the experiment.

## Supplementary Information

**Additional file 1: Figure S1.** Trypan blue staining of wild-type and *nrt1.5* roots. The 4-d-old seedlings were transferred onto LK and MS medium for 3 days. The roots were stained with 0.4% Trypan for 5 min and photographed under a dissecting microscope (Nikon) equipped with a Canon DSLR camera. Bars = 0.1 mm. **Figure S2.** The transcript of *PIN2* and *PIN3* in wild-type and *nrt1.5* mutants under LK stress. The 4-d-old seedlings were transferred to LK and MS medium for 3 days. The roots were collected for analysis the transcript of *PIN2* and *PIN3*. **Figure S3.** A more-detailed view of PIN2-GFP in wild-type and *nrt1.5* mutant under LK stress. The *ProPIN2:PIN2:GFP* crossing lines were transferred to LK medium for 3 h and 6 h. The GFP fluorescence in plants roots was observed. Bars = 10 μm. **Figure S4.** Analysis the PIN3 in wild-type and *nrt1.5* mutant under LK stress. The *ProPIN3:PIN3:GFP* crossing lines were transferred to LK medium for 3 d. The GFP fluorescence in plants roots was observed. Bar = 50 μm.

## Data Availability

All data supporting the findings of this study are available within the paper and within its supplementary data published online.

## Abbreviations

LK: Low potassium
NRT1.5: Nitrate Transporter 1.5
PIN1: PIN-FORMED 1
PIN2: PIN-FORMED 2
PIN3: PIN-FORMED 3
PIN7: PIN-FORMED 7
IAA: Indole-3-acetic acid
IBA: Indole-3-butyric acid
NAA: 1-naphthlcetic acid
NPA: Naphthylphthalamic acid
KUP/HAK: High-affinity K^+^ transporter/K^+^ uptake permease
PM: Plasma membrane
AUX1: AUXIN1
AXR1: AUXIN RESISTANT1
AXR2: AUXIN RESISTANT2
LP: Low phosphorus
LR: Lateral Root
LKS2: Low K^+^ Sensitive 2
PCR: Polymerase Chain Reaction
GFP: Green Fluorescent Protein
MS: Murashige and Skoog
MZ: Meristem zone
EZ: Elongation zone

## References

[1] Forde BG, Lorenzo H (2001). The nutritional control of root development. Plant Soil.

[2] López-Bucio J, Cruz-Ramírez A, Herrera-Estrella L (2003). The role of nutrient availability in regulating root architecture. Curr Opin Plant Biol.

[3] López-Bucio J, Hernández-Abreu E, Sánchez-Calderón L, Nieto-Jacobo MF, Simpson J, Herrera-Estrella L (2002). Phosphate availability alters architecture and causes changes in hormone sensitivity in the Arabidopsis root system. Plant Physiol.

[4] Sánchez-Calderón L, López-Bucio J, Chacón-López A, Cruz-Ramírez A, Nieto-Jacobo F, Dubrovsky JG (2005). Phosphate starvation induces a determinate developmental program in the roots of Arabidopsis thaliana. Plant Cell Physiol.

[5] Williamson LC, Ribrioux SP, Fitter AH, Leyser HM (2001). Phosphate availability regulates root system architecture in Arabidopsis. Plant Physiol.

[6] Linkohr BI, Williamson LC, Fitter AH, Leyser HM (2002). Nitrate and phosphate availability and distribution have different effects on root system architecture of Arabidopsis. Plant J.

[7] Clarkson DT, Hanson JB (1980). The mineral nutrition of higher plants. Annual Review of Plant Physiol.

[8] Maathuis FJ (2009). Physiological functions of mineral macronutrients. Curr Opin Plant Biol.

[9] Schroeder JI, Ward JM, Gassmann W (1994). Perspectives on the physiology and structure of inward-rectifying K^+^ channels in higher plants: biophysical implications for K^+^ uptake. Annu Rev Biophys Biomol Struct.

[10] Giehl RF, Gruber BD, von Wirén N (2014). It's time to make changes: modulation of root system architecture by nutrient signals. J Exp Bot.

[11] Jung JY, Shin R, Schachtman DP (2009). Ethylene mediates response and tolerance to potassium deprivation in Arabidopsis. Plant Cell.

[12] Kellermeier F, Chardon F, Amtmann A (2013). Natural variation of Arabidopsis root architecture reveals complementing adaptive strategies to potassium starvation. Plant Physiol.

[13] Drew MC (1975). Comparison of effects of a localized supply of phosphate, nitrate, ammonium and potassium on growth of seminal root system, and shoot, in barley. New Phytol.

[14] Armengaud P, Breitling R, Amtmann A (2004). The potassium-dependent transcriptome of Arabidopsis reveals a prominent role of jasmonic acid in nutrient signaling. Plant Physiol.

[15] Chérel I, Lefoulon C, Boeglin M, Sentenac H (2014). Molecular mechanisms involved in plant adaptation to low K^+^ availability. J Exp Bot.

[16] Wang Y, Wu WH (2013). Potassium transport and signaling in higher plants. Annu Rev Plant Biol.

[17] Shin R, Schachtman DP (2004). Hydrogen peroxide mediates plant root cell response to nutrient deprivation. Proc Natl Acad Sci U S A.

[18] Osakabe Y, Arinaga N, Umezawa T, Katsura S, Nagamachi K, Tanaka H (2013). Osmotic stress responses and plant growth controlled by potassium transporters in Arabidopsis. Plant Cell.

[19] Rigas S, Debrosses G, Haralampidis K, Vicente-Agullo F, Feldmann KA, Grabov A (2001). TRH1 encodes a potassium transporter required for tip growth in Arabidopsis root hairs. Plant Cell.

[20] Vicente-Agullo F, Rigas S, Desbrosses G, Dolan L, Hatzopoulos P, Grabov A (2004). Potassium carrier TRH1 is required for auxin transport in *Arabidopsis* roots. Plant J.

[21] Zhang ML, Huang PP, Ji Y, Wang S, Wang SS, Li Z, Guo Y, Ding Z, Wu WH, Wang Y (2020). KUP9 maintains root meristem activity by regulating K^+^ and auxin homeostasis in response to low K. EMBO Rep.

[22] Rigas S, Ditengou FA, Ljung K, Daras G, Tietz O, Palme K (2013). Root gravitropism and root hair development constitute coupled developmental responses regulated by auxin homeostasis in the Arabidopsis root apex. New Phytol.

[23] Cao Y, Glass AD, Crawford NM (1993). Ammonium inhibition of Arabidopsis root growth can be reversed by potassium and by auxin resistance mutations aux1, axr1, and axr2. Plant Physiol.

[24] Friml J, Wiśniewska J, Benková E, Mendgen K, Palme K (2002). Lateral relocation of auxin efflux regulator PIN3 mediates tropism in Arabidopsis. Nature.

[25] Müller A, Guan C, Gälweiler L, Tänzler P, Huijser P, Marchant A (1998). AtPIN2 defines a locus of Arabidopsis for root gravitropism control. EMBO J.

[26] Friml J, Benková E, Mayer U, Palme K, Muster G (2003). Automated whole mount localisation techniques for plant seedlings. Plant J.

[27] Dolan L (2013). Pointing PINs in the right directions: a potassium transporter is required for the polar localization of auxin efflux carriers. New Phytol.

[28] Li J, Wu WH, Wang Y (2017). Potassium channel AKT1 is involved in the auxin-mediated root growth inhibition in Arabidopsis response to low K^+^ stress. J Integr Plant Biol.

[29] Jing Y, Zheng X, Zhang D, Shen N, Wang Y, Yang L (2019). Danger-associated peptides interact with PIN-dependent local auxin distribution to inhibit root growth in Arabidopsis. Plant Cell.

[30] Lin DL, Yao HY, Jia LH, Tan JF, Xu ZH, Zheng WM (2020). Phospholipase D-derived phosphatidic acid promotes root hair development under phosphorus deficiency by suppressing vacuolar degradation of PIN-FORMED2. New Phytol.

[31] Lin SH, Kuo HF, Canivenc G, Lin CS, Lepetit M, Hsu PK (2008). Mutation of the Arabidopsis NRT1.5 nitrate transporter causes defective root-to-shoot nitrate transport. Plant Cell.

[32] Li H, Yu M, Du XQ, Wang ZF, Wu WH, Quintero FJ (2017). NRT1.5/NPF7.3 functions as a proton-coupled H^+^/K^+^ antiporter for K^+^ loading into the xylem in Arabidopsis. Plant Cell.

[33] Watanabe S, Takahashi N, Kanno Y, Suzuki H, Aoi Y, Takeda-Kamiya N (2020). The *Arabidopsis* NRT1/PTR FAMILY protein NPF7.3/NRT1.5 is an indole-3-butyric acid transporter involved in root gravitropism. Proc Natl Acad Sci U S A.

[34] Tal I, Zhang Y, Jørgensen ME, Pisanty O, Barbosa IC, Zourelidou M (2016). The Arabidopsis NPF3 protein is a GA transporter. Nat Commun.

[35] Kanno Y, Kamiya Y, Seo M (2013). Nitrate does not compete with abscisic acid as a substrate of AtNPF4.6/NRT1.2/AIT1 in Arabidopsis. Plant Signal Behav.

[36] Krouk G, Lacombe B, Bielach A, Perrine-Walker F, Malinska K, Mounier E (2010). Nitrate-regulated auxin transport by NRT1.1 defines a mechanism for nutrient sensing in plants. Dev Cell.

[37] Ding Z, Friml J (2010). Auxin regulates distal stem cell differentiation in Arabidopsis roots. Proc Natl Acad Sci U S A.

[38] Moubayidin L, Di Mambro R, Sozzani R, Pacifici E, Salvi E, Terpstra I (2013). Spatial coordination between stem cell activity and cell differentiation in the root meristem. Dev Cell.

[39] Stepanova AN, Yun J, Likhacheva AV, Alonso JM (2007). Multilevel interactions between ethylene and auxin in Arabidopsis roots. Plant Cell.

[40] Blakeslee JJ, Peer WA, Murphy AS (2005). Auxin transport. Curr Opin Plant Biol.

[41] Blilou I, Xu J, Wildwater M, Willemsen V, Paponov I, Friml J (2005). The PIN auxin efflux facilitator network controls growth and patterning in Arabidopsis roots. Nature.

[42] Wisniewska J, Xu J, Seifertová D, Brewer PB, Ruzicka K, Blilou I (2006). Polar PIN localization directs auxin flow in plants. Science.

[43] Petrásek J, Friml J (2009). Auxin transport routes in plant development. Development.

[44] Pan J, Fujioka S, Peng J, Chen J, Li G, Chen R (2009). The E3 ubiquitin ligase SCFTIR1/AFB and membrane sterols play key roles in auxin regulation of endocytosis, recycling, and plasma membrane accumulation of the auxin efflux transporter PIN2 in Arabidopsis thaliana. Plant Cell.

[45] Chen CZ, Lv XF, Li JY, Yi HY, Gong JM (2012). Arabidopsis NRT1.5 is another essential component in the regulation of nitrate reallocation and stress tolerance. Plant Physiol.

[46] Meng S, Peng JS, He YN, Zhang GB, Yi HY, Fu YL (2016). Arabidopsis NRT1.5 mediates the suppression of nitrate starvation-induced leaf senescence by modulating foliar potassium level. Mol Plant.

[47] Kleine-Vehn J, Leitner J, Zwiewka M, Sauer M, Abas L, Luschnig C (2008). Differential degradation of PIN2 auxin efflux carrier by retromer-dependent vacuolar targeting. Proc Natl Acad Sci U S A.

[48] Vega A, Fredes I, O'Brien J, Shen Z, Ötvös K, Abualia R, Benkova E, Briggs SP, Gutiérrez RA (2021). Nitrate triggered phosphoproteome changes and a PIN2 phosphosite modulating root system architecture. EMBO Rep.

[49] Xu J, Scheres B (2005). Dissection of Arabidopsis ADP-RIBOSYLATION FACTOR 1 function in epidermal cell polarity. Plant Cell.

[50] Xu J, Li HD, Chen LQ, Wang Y, Liu LL, He L (2006). A protein kinase, interacting with two calcineurin B-like proteins, regulates K^+^ transporter AKT1 in Arabidopsis. Cell.

