# Supplementary material for: Transporter NRT1.5/NPF7.3 suppresses primary root growth under low K+ stress by regulating the degradation of PIN-FORMED2
Source: BMC Plant Biol. 2022 Jul 8;22:330. doi: 10.1186/s12870-022-03730-6 (PMC9264542; doi:10.1186/s12870-022-03730-6)
Supplement: Supplementary file 1 — Additional file 1: Figure S1. Trypan blue staining of wild-type and nrt1.5 roots. The 4-d-old seedlings were transferred onto LK and MS medium for 3 days. The roots were stained with 0.4% Trypan for 5 min and photographed under a dissecting microscope (Nikon) equipped with a Canon DSLR camera. Bars = 0.1 mm. Figure S2. The transcript of PIN2 and PIN3 in wild-type and nrt1.5 mutants under LK stress. The 4-d-old seedlings were transferred to LK and MS medium for 3 days. The roots were collected for analysis the transcript of PIN2 and PIN3. Figure S3. A more-detailed view of PIN2-GFP in wild-type and nrt1.5 mutant under LK stress. The ProPIN2:PIN2:GFP crossing lines were transferred to LK medium for 3 h and 6 h. The GFP fluorescence in plants roots was observed. Bars = 10 μm. Figure S4. Analysis the PIN3 in wild-type and nrt1.5 mutant under LK stress. The ProPIN3:PIN3:GFP crossing lines were transferred to LK medium for 3 d. The GFP fluorescence in plants roots was observed. Bar = 50 μm. [file 12870_2022_3730_MOESM1_ESM.docx]

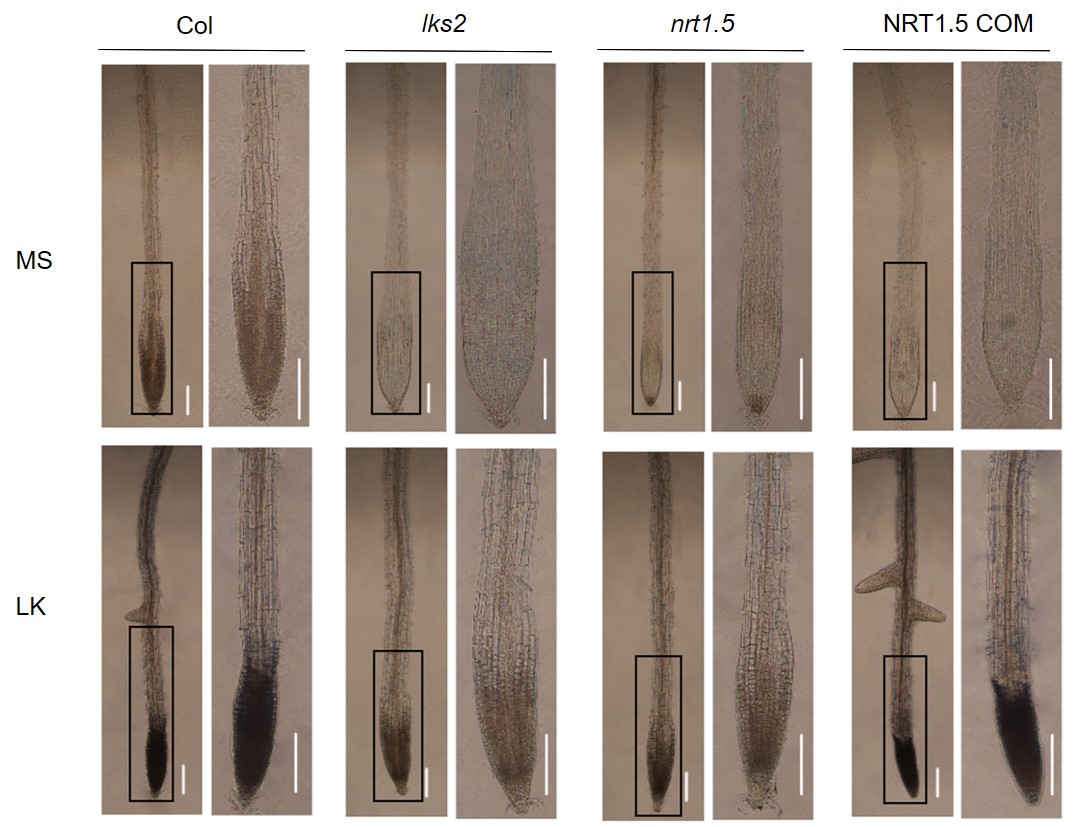


Figure S1 Trypan blue staining of wild-type and *nrt1.5* roots.

The 4-d-old seedlings were transferred onto LK and MS medium for 3 days. The roots were stained with 0.4% Trypan for 5 min and photographed under a dissecting microscope (Nikon) equipped with a Canon DSLR camera. Bars=0.1 mm.


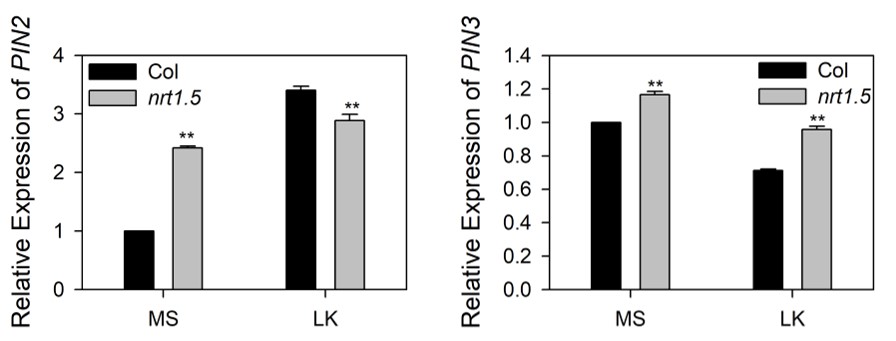


Figure S2 The transcript of *PIN2* and *PIN3* in wild-type and *nrt1.5* mutants under LK stress.

The 4-d-old seedlings were transferred to LK and MS medium for 3 days. The roots were collected for analysis the transcript of *PIN2* and *PIN3*.


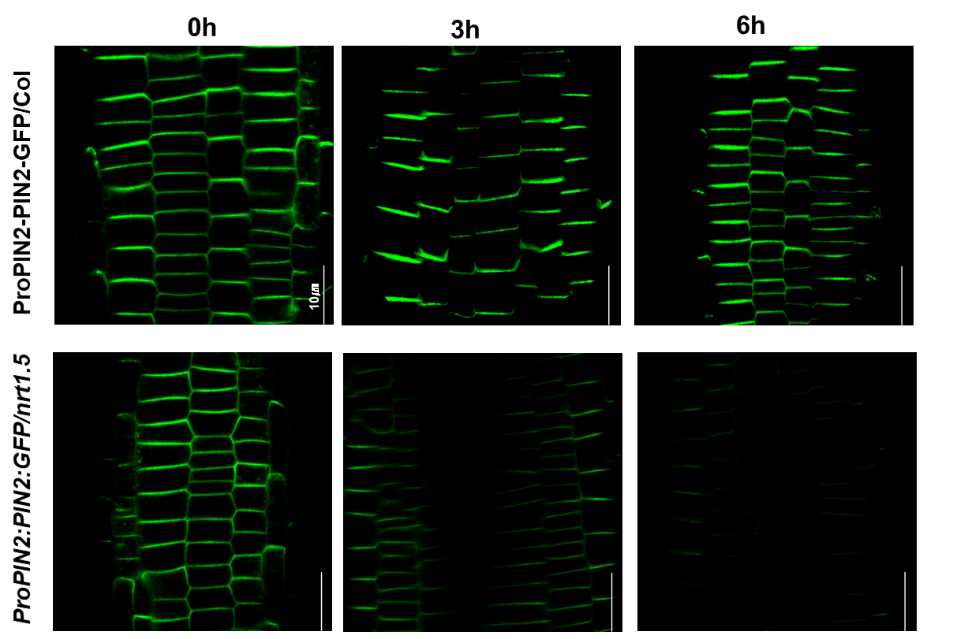


Figure S3 A more-detailed view of PIN2-GFP in wild-type and *nrt1.5* mutant under LK stress.

The *ProPIN2:PIN2:GFP* crossing lines were transferred to LK medium for 3 h and 6 h. The GFP fluorescence in plants roots was observed. Bar=10 μm.


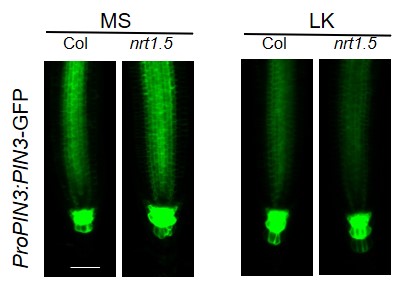


Figure S4 Analysis the PIN3 in wild-type and *nrt1.5* mutant under LK stress.

The *ProPIN3:PIN3:GFP* crossing lines were transferred to LK medium for 3 d. The GFP fluorescence in plants roots was observed. Bar=50 μm.
